# Brief Reports: Influence of Friendship on Loneliness Among Adolescents with Autism Spectrum Disorders in Japan

**DOI:** 10.1007/s10803-023-05958-z

**Published:** 2023-04-06

**Authors:** Motofumi Sumiya, Atsushi Senju

**Affiliations:** 1 Research Center for Child Mental Development, Hamamatsu University School of Medicine, Hamamatsu, Japan; 2 United Graduate School of Child Development, School of Medicine, Osaka University, Kanazawa University, Hamamatsu University, Chiba University and University of Fukui, Osaka/Kanazawa/Hamamatsu/Chiba/Fukui, Osaka, Japan

**Keywords:** Adolescents, Autism Spectrum Disorder, Friendship, Loneliness

## Abstract

**Purpose:**

Previous studies have reported that people with autism spectrum disorder (ASD) have higher levels of loneliness than neurotypical (NTP) people, most likely because of their difficulties in social communication with their predominantly NTP peers. However, direct investigations on the causal influence of friendship on their feelings of loneliness is scarce.

**Methods:**

In the current study, using the causal mediation analysis, we investigated whether friendship among ASD individuals influences their feelings of loneliness, especially during adolescence when the importance of friendship is typically most elevated. Furthermore, we examined whether individual differences in autistic behavioral features or age affect feelings of loneliness or the qualities of friendship with linear regression analyses.

**Results:**

The results demonstrated that the higher levels of loneliness in adolescents with ASD were mediated by one aspect of friendship, companionship. We also found that positive aspects of friendship, but not negative aspects, influence the feelings of loneliness in both ASD and NTP populations. One subcategory of the measured autistic trait, difficulty in imagination, which is associated the ability to consider another’s perspective, had a negative relationship with the positive aspects of friendship in the ASD group, but not in the NTP group.

**Conclusion:**

These findings indicate that the quality of the positive aspects of friendship is similarly important for both adolescents with ASD and NTP adolescents, but the autistic behavioral features could interfere with the experience of such positive friendships.

Loneliness is defined as an adverse experience that affects an individual’s social, emotional, and cognitive functioning due to difficulties in their social relationships (Lasgaard et al., 2010). Previous studies using both quantitative and qualitative methodologies reported that people with autism spectrum disorder (ASD), who are known to have difficulties in social communication and repetitive patterns of behavior (American Psychiatric Association, 2013), have high levels of loneliness from childhood to adulthood (e.g., Bauminger et al., 2003; Sumiya et al., 2018; White & Roberson-Nay, 2009). A recent meta-analysis, which included 23 quantitative studies that investigated the differences in loneliness levels between ASD and neurotypical (NTP) individuals, found increased loneliness in the ASD population from childhood to adulthood (Hymas et al., 2022). Similarly, a recent systematic review that targeted qualitative, quantitative, and mixed-methods studies on loneliness in adults with ASD explored their heightened loneliness and desire for social connection (Umagami et al., 2022). Although these studies have consistently highlighted the high level of loneliness among the ASD population, only a limited number of studies have investigated factors or characteristics that causally influence feelings of loneliness in people with ASD.

One main factor that is known to influence the feeling of loneliness is friendship. According to a meta-analysis of friendships among school-age boys with ASD, compared to their NTP peers, their number of friends is low, and the qualities of friendship among ASD boys is not as optimal as among their NTP peers in many aspects, such as companionship, help, security, and closeness, but it is comparable in regard to conflict (Mendelson et al., 2016). Similarly, a recent study targeting 306 ASD and NTP adolescents reported that those with ASD have lower levels of positive friendship qualities such as companionship or support, but no differences were found in negative friendship qualities such as conflict or betrayal (O’Connor et al., 2022). These findings indicate that children and adolescents with ASD are aware of situations in which they do not emotionally and socially connect with friends, especially in positive aspects. Therefore, the causal relationship between such diverse aspects of friendship such as companionship, help, security, closeness and conflict and feelings of loneliness at these ages, particularly during adolescence when the importance of friendship is typically most elevated (Blakemore, 2019), needs to be explored.

Furthermore, many in the ASD individuals evaluate friendship as important. A recent study that synthesized the findings of qualitative studies explored the various experiences of friendship in ASD individuals, such as the benefits of having friends for emotional support (Black et al., 2022); however, this study also noted divergent individual desires for friendship, including enjoyment of being alone or having many friends, possibly related to individual differences in coping with the demand of friendship and social relationship. Accordingly, studies are needed to explore how the individual difference in behavioral features associated with autism (social skills, change, attention, communication, and imagination), age, and sex affect their different aspects of friendship and feelings of loneliness.

These preceding studies strongly indicated the influence of low-quality friendship in the ASD adolescents on their heightened levels of loneliness, but the investigations on the causal relationship between these two parameters, as well as the influence of different aspects of friendships, are scarce. Therefore, the current study aims to examine (1) whether friendship perspectives such as companionship, help, security, closeness, and conflict mediate the feelings of loneliness, which are differ in ASD and NTP adolescents, (2) whether individual characteristics such as behavioral features associated with autism influence their friendship quality and loneliness. The current study is one of the first to investigate the factors associated with loneliness and friendship in adolescents with ASD in Japan, so it adds to the body of studies on these topics, which has primarily included research participants from Western Europe and North America.

## Methods

### Participants

Sixteen adolescents with ASD aged 10–15 years (6 females; mean age = 12.25 years; standard deviation [SD] = 1.53 years) and 25 NTP adolescents aged 10–15 years (11 females; mean age = 12.24 years; SD = 1.42 years) participated in the study. These participants were recruited from several institutes with which the first author was directly or indirectly associated. In the ASD group, we targeted the participants whose full IQ on the WISC- III was above 80. There were no significant differences between the two groups of participants in their numbers by sex (Χ^2^ = 0.008, p = 0.931) or age (t = 0.021, p = 0.983). This study was conducted at Shirayuri University, where the first author was affiliated at the time of data collection. Since the college did not have a specific institutional review board or an ethics committee, the study was reviewed and approved by an ad hoc committee called the “Provisional Ethics Committee,” which consisted of a group of developmental psychologists at the college, and by the directors of the centers and schools from which students were recruited. Participation in this study was voluntary, and before the study, all participants and their parents signed informed consent documents according to the principles outlined in the Declaration of Helsinki. To confirm the participants’ clinical manifestation and cognitive profiles associated with ASD, the Japanese version of the Autism Spectrum Quotient (AQ) (Wakabayashi et al., 2007) was administered to their parents. Further, parents of adolescents with ASD were asked to provide their child’s WISC-III results from within the past 3 years. These data are summarized in Table S1. More details are included in the supplemental files.

### Self-Report Questionnaires

In the current study, we asked ASD and NTP adolescents to complete multiple questionnaires. We used a modified version of the Loneliness Scale for ASD (Asher & Wheeler, 1985; Bauminger et al., 2003) to measure emotional loneliness (EL) and social loneliness (SL), and global loneliness (GL) as a combination of these measures. The modified version of the Loneliness Scale consists of 30 statements, 13 items for the SL (e.g., “I have many friends in class”) subscale and 9 items for the EL (e.g., “I feel very sad in class”) and 8 filler items. We also used the Friendship Qualities Scale (Bukowski et al., 1994), which consist of 23 items that examines five aspects of friendship quality: (a) companionship, such as time spent together voluntarily (e.g., “My friend and I spend all our free time together”); (b) help, including both assistance and protection from persecution (e.g., “My friend would help me if I needed it”); (c) security, including the idea that the relationship transcends certain issues and trust (e.g., “If there is something bothering me I can tell my friend about it even if it is something I cannot tell to other people”); (d) closeness, consisting of both the child’s feelings toward the partner and his/her perception of the partner’s feelings (e.g., “I think about my friend even when my friend is not around”); and (e) conflict, disagreement in a relationship with a friend (e.g., “My friend and I can argue a lot”). To analyze a subscale, conflict, we reversed the scores. The first author and a third-party translator translated both questionnaires. Both questionnaires presented adequate internal consistencies as previous studies (GL of Loneliness Scale: Cronbach’s α of 0.93 in Bauminger et al. 2003, and 0.92 in the current study, Friendship Qualities Scale subscales: Cronbach’s α between 0.57 and 0.86 in Bauminger et al. 2010, and between 0.61 and 0.85 in the current study). These questionnaires are administered in the participants’ homes or at the institutions where they are recruited. Some participants receive assistance in completing the questionnaires from their caregivers or teachers. More details on this section are included in the supplemental files.

### Statistical Analyses

#### Between Groups

All statistical analyses were performed using R software (version 4.2.0). We first conducted linear regression analyses to investigate the influence of group differences on the perception of loneliness (GL, EL, SL) and the qualities of friendship (companionship, conflict, help, security, closeness). Models were fitted using the lm_robust command from the “estimatr” package (Blair et al., 2018) using heteroskedasticity-consistent (HC) 2 robust standard errors. In these analyses, a categorical variable was coded as (− 0.5 = ASD, 0.5 = NTP) for the group. For the calculation of effect size (Cohen’s f^2^) on each regressor, we used “effectsize” package (Ben-Shachar et al., 2020). Next, we conducted the causal mediation analysis using “mediation” package (Tingley et al., 2014) to fit the single mediator model using robust standard errors to protect against heteroskedasticity. The model consists of regression equations for the direct effect, from the determinant variable to the mediators, and from the mediators and determinant variable to the outcome variable. Consequently, we performed sensitivity analysis and post hoc power analysis to determine the power of the current analysis. Details and results of these analyses are provided in the supplementary file.

#### Within Groups

Within each group, we used multiple linear regression analyses to investigate factors that influence their perceptions of loneliness and the qualities of friendship. Following O’Connor et al. (2022), we summarized companionship, security, help, and closeness as positive aspects of friendship (FQp) and conflict as a negative aspect of friendship (FQn), to decrease the number of correlated regressors. We conducted these analyses with a consideration of correlation among potential factors (Age, AQ, verbal IQ, FQp, FQn, Sex) (Figures S1 & S2). We confirmed the multicollinearity of regressors within a model using the performance package (Lüdecke et al., 2021). First, we investigated whether the feeling of loneliness is influenced by the qualities of friendship in each group. As confounding covariates, these models included age and sex, and in the ASD group on, verbal IQ. Then, we investigated whether feelings of loneliness and the qualities of friendship are influenced by behavioral features associated with autism (e.g., difficulties on communication or imagination). In these analyses, the categorical variable was coded as (− 0.5 = female, 0.5 = male) for the sex difference.

## Results

### Between Groups

The results of the single linear regression analyses on the perception of loneliness and the qualities of friendship revealed that adolescents with ASD feel significantly higher levels of loneliness than NTP concerning EL (β= -0.629, p = 0.025), SL (β= -0.5956, p = 0.019) and GL (β= -0.601, p = 0.020). On the qualities of friendship, we found that ASD adolescents reported significantly lower levels of companionship (β = 0.539, p = 0.020) than NTP, but not on the other perspectives (conflict: β = 0.008, p = 0.984; help: β = 0.400, p = 0.112; security: β = 0.285 p = 0.243; closeness: β = 0.074, p = 0.738) (Figure S3). As there was no differential tendency between EL and SL (Figure S4), which was confirmed by two-way ANOVA (the main effect of group: F(1.39) = 8.286, p = 0.007, η^2^_p_ **=** 0.175; the main effects of type: F(1.39) = 9.723, p = 0.003, η^2^_p_ = 0.200) and interaction: F(1.39) = 0.057, p = 0.8133, η^2^_p_ = 0.001), we focused on GL, which is the global score incorporating both EL and SL, for further analysis. The distribution of each report is summarized in Figure S5.

**Fig. 1:**
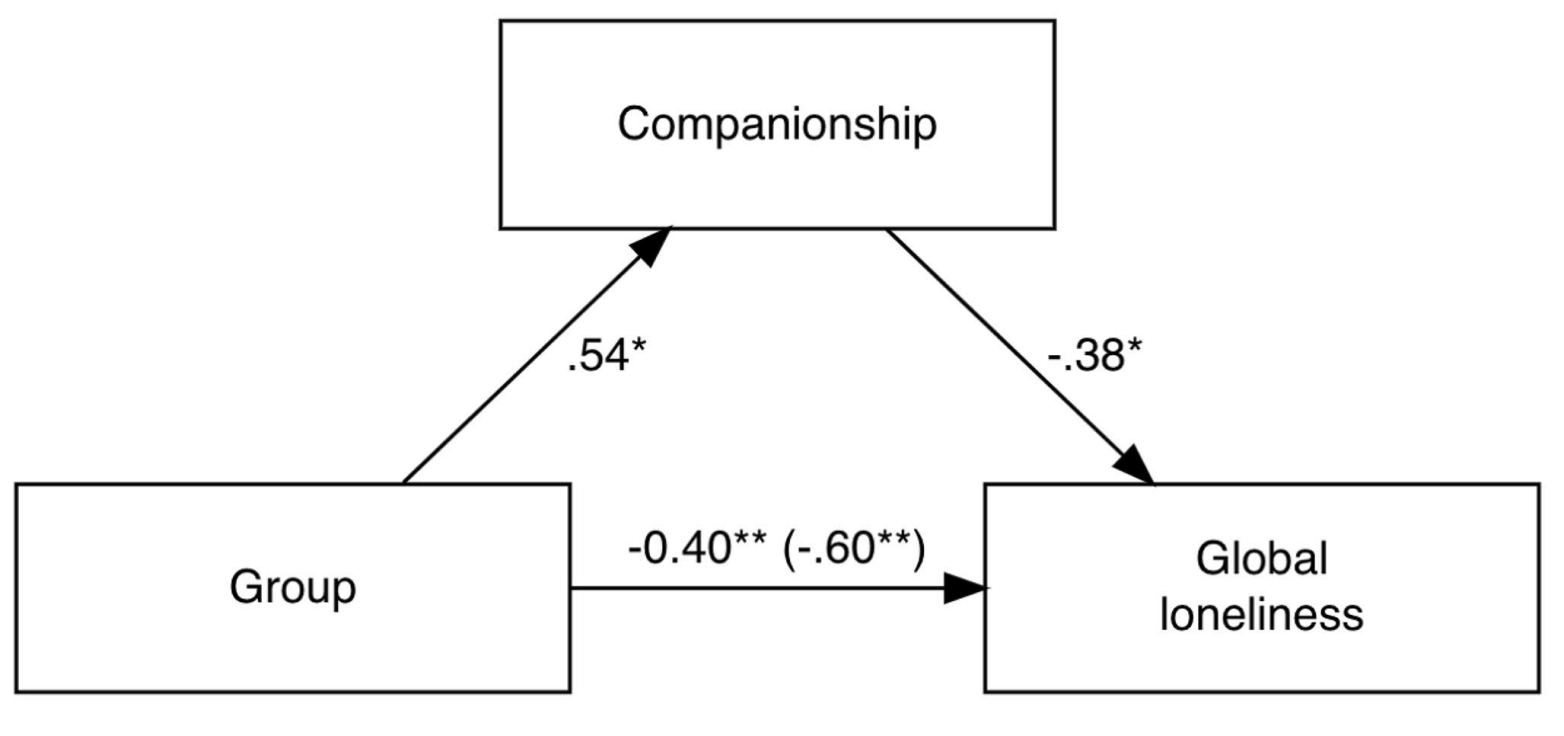
Conceptual Diagram of Causal Mediation Analysis of the Relationship between the Group Difference, Companionship, and Global Loneliness

With the above in mind, we targeted the mediation effect of companionship on the group differences in GL. The results of the causal mediation analysis showed that the effects of group differences on GL were fully mediated via companionship. As Fig. 1 illustrates, the regression coefficient between the group differences and GL and the regression coefficient between companionship and GL were both significant. The indirect effect was (0.5388)*(-0.3780) = -0.204. We tested the significance of this indirect effect using bootstrapping procedures. Unstandardized indirect effects were computed for each of the 10,000 bootstrapped samples, and the 95% confidence interval was computed by determining the indirect effects at the 2.5 and 97.5 percentiles. The bootstrapped unstandardized indirect effect was − 0.204, and the 95% confidence interval ranged from − 0.524 to -0.05. Thus, the indirect effect was statistically significant (p = 0.014).

### Within Groups

**Fig. 2:**
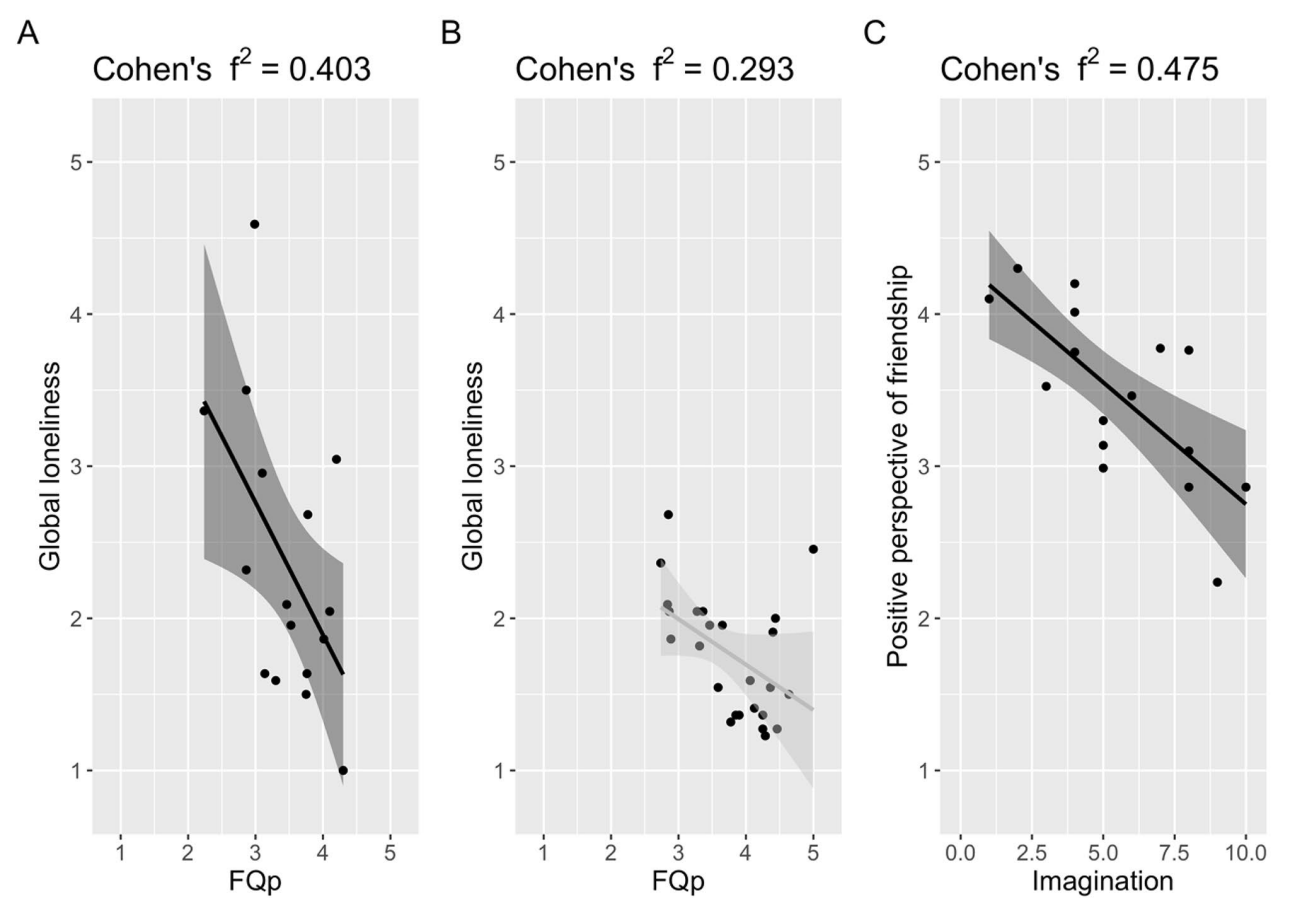
(A) Influence of Positive Friendship on Global Loneliness in the ASD Group, (B) in the NTP Group. and (C) Influence of an Autistic Trait, Imagination, on Positive Friendship in the ASD Group

We investigated whether the qualities of friendship influence feelings of loneliness (GL) with a regression model within each group. In the ASD group, the overall regression was statistically significant (adjusted R^2^ = 0.573, F(5, 10) = 7.746, p = 0.003). The qualities of positive friendship significantly predicted the feeling of loneliness (β = -0.847, p = 0.007) (Fig. 2A), but not the qualities of negative friendship (β = 0.1076, p = 0.361). We also found that higher age (β = 0.207, p = 0.045) and being female (β = -0.819, p = 0.026) predicted higher levels of loneliness, but not verbal IQ (β = -0.010, p = 0.256). Similarly, in the NTP group, the overall regression was statistically significant (adjusted R^2^ = 0.309, F(4, 30) = 3.453, p = 0.027). The qualities of positive friendship significantly predicted feelings of loneliness (β = -0.4022, p = 0.013) (Fig. 2B), but not the qualities of negative friendship (β = 0.1723, p = 0.1420), age (β = -0.082, p = 0.217) or sex differences (β = -0.018, p = 0.911).

We further investigated whether autistic characteristics influence feelings of loneliness and the qualities of friendship. First, the overall regression for GL was not statistically significant in the ASD group (adjusted R^2^ = 0.078, F(5, 10) = 3.104, p = 0.060) or in the NTP group (adjusted R^2^ = -0.003, F(5, 19) = 1.136, p = 0.376). We found no association between GL and each component of AQ in either group. Second, the overall regression for the qualities of friendship were statistically significant in the ASD group (FQp: adjusted R^2^ = 0.333, F(5, 10) = 7.746, p = 0.008, FQn; adjusted R^2^ = 0.236, F(5, 10) = 7.746, p = 0.008). Imagination (β = -0.1456, p = 0.011) significantly predicted a positive aspect of friendship (Fig. 2C), but not social skills (β = -0.006, p = 0.914), change (β = -0.012, p = 0.914), attention (β = -0.033, p = 0.667), or communication (β =-0.075, p = 0.548). Furthermore, social skills (β = 0.309, p = 0.008) significantly predicted negative aspects of friendship, but not change (β = -0.1395, p = 0.462), attention (β = 0.1143, p = 0.453), communication (β =-0.3373, p = 0.283), or imagination (β = 0.1169, p = 0.395). These associations with loneliness and the subcomponents of AQ were not found in the NTP group for positive (adjusted R^2^ = 0.127, F(5, 19) = 3.526, p = 0.020) or negative friendship qualities (adjusted R^2^ = -0.177, F(5, 19) = 0.640, p = 0.672).

The parameters and distribution of each report are summarized in Figures S7–S14 and Tables S2–S4. For all analyses, we calculated the multicollinearity of regressors and confirmed the low correlation of that predictor with other predictors (Variance Inflation Factor < 5) (Figures S15–S18).

## Discussion

To the best of the authors’ knowledge, this is the first study to demonstrate that higher levels of loneliness in adolescents with ASD are mediated by one aspect of friendship, companionship. Companionship, as measured by the Friendship Qualities Scale, represents the frequency and time spent together in common activities and playing with friends. It has been known that autistic children are less likely than NTP children to participate in social activities with friends and are more likely to play video and computer games by themselves (Shane & Albert, 2008; Solish et al., 2010). Therefore, our findings indicate that time spent together in play is a critical factor concerning feelings of loneliness, which include affective and social loneliness, for autistic adolescents. Although previous qualitative studies have explored the various forms of social motivation in individuals with ASD (Black et al., 2022; Sumiya et al., 2018), our findings strongly indicate that many of them feel lonely because of the difficulties they experience when spending time and playing and seeking more social interactions with their friends.

Next, we considered who feels lonelier, and what kinds of autistic behavioral features affect their friendships. First, we found that positive aspects of friendship, not negative aspects, influence feelings of loneliness in both the ASD and NTP groups. These results support the above findings on group differences and further emphasize that, regardless of an ASD diagnosis, adolescents highly value the emotional and physical aspects of relationships with their friends. Second, we found that one of the autistic behavioral features, difficulty in imagination, which is associated with the ability to consider another person’s perspective, is associated with lower positive aspects of friendship only in the ASD group. We also found that feelings of loneliness increase with age in the ASD group. It is known that adolescence is a period where more advanced skills of perspective-taking are required to meet increasingly complex social interactions (Hollarek & Lee, 2022). Accordingly, our findings indicate that the qualities of the positive aspects of friendship are similarly important for both ASD and NTP adolescents, but some autistic behavioral features could negatively affect the experience of these positive friendships.

The current study has some limitations. The number of participants was limited in this study due to the challenges in recruitment, especially for within-group analyses. Thus, we did not conduct a mediation analysis in each group or interpret the results in relation to sex differences. Future studies should continue to focus on the causal relationship between loneliness and friendship among adolescents with ASD using a larger sample, especially from the Asian population, to consider the nature of friendship in a specific cultural context. In addition, in this study, we used questionnaires that were translated for this study and therefore not standardized in the Japanese population prior to the current study. Also, some subscales of the Friendship Qualities Scale were not high enough, as in a previous study (Bauminger et al., 2010), suggesting the need for a friendship questionnaire for the ASD population.

To conclude, the current study is the first to demonstrate that higher levels of loneliness in adolescents with ASD are mediated by companionship, one aspect of friendship which relates to the frequency and time spent together in common activities and playing with friends. This result empirically confirmed the association between friendships and loneliness in ASD, which has been indicated from studies in North America and Europe, in Japanese adolescence. It suggests that friendships are an important mediating factor to alleviate loneliness for ASD adolescence in Eastern Asian cultures as well. Future studies would be fruitful to explore multiple aspects of friendship, including companionship, and its relationship with loneliness in ASD adolescents belonging to a wider range of cultural backgrounds. Such understanding will help to improve the quality of life for ASD adolescents globally, which would be catered to the cultural environment in which they live.
